# Mechanical Properties of Repaired Welded Pipe Joints Made of Heat-Resistant Steel P92

**DOI:** 10.3390/ma18122908

**Published:** 2025-06-19

**Authors:** Filip Vučetić, Branislav Đorđević, Dorin Radu, Stefan Dikić, Lazar Jeremić, Nikola Milovanović, Aleksandar Sedmak

**Affiliations:** 1 Innovation Center of Faculty of Mechanical Engineering, Kraljice Marije 16, 11120 Belgrade, Serbia; fvucetic@mas.bg.ac.rs (F.V.); brdjordjevic@mas.bg.ac.rs (B.Đ.); ljeremic@mas.bg.ac.rs (L.J.); nmilovanovic@mas.bg.ac.rs (N.M.); 2 Faculty of Civil Engineering, Transilvania University of Brașov, Turnului Street 5, 500152 Brașov, Romania; 3 Faculty of Technology and Metallurgy, University of Belgrade, Karnegijeva 4, 11120 Belgrade, Serbia; sdikic@tmf.bg.ac.rs; 4 Faculty of Mechanical Engineering, University of Belgrade, Kraljice Marije 16, 11120 Belgrade, Serbia; asedmak@mas.bg.ac.rs

**Keywords:** heat-resistant steel P92, welding, heat input, mechanical properties, microstructural analysis, weld metal, heat-affected zone

## Abstract

This research provides a detailed investigation into the mechanical properties and microstructural evolution of heat-resistant steel P92 subjected to both initial (i) welding procedures and simulated (ii) repair welding. The study addresses the influence of critical welding parameters, including preheating temperature, heat input, and post-weld heat treatment (PWHT), with a particular emphasis on the metallurgical consequences arising from the application of repair welding thermal cycles. Through the analysis of three welding probes—initially welded pipes using the PF (vertical upwards) and PC (horizontal–vertical) welding positions, and a PF-welded pipe undergoing a simulated repair welding (also in the PF position)—the research compares microstructure in the parent material (PM), weld metal (WM), and heat-affected zone (HAZ). Recognizing the practical limitations and challenges associated with achieving complete removal of the original WM under the limited (in-field) repair welding, this study provides a comprehensive comparative analysis of uniaxial tensile properties, impact toughness evaluated via Charpy V-notch testing, and microhardness measurements conducted at room temperature. Furthermore, the research critically analyzes the influence of the complex thermal cycles experienced during both the initial welding and repair welding procedures to elucidate the practical application limits of this high-alloyed, heat-resistant P92 steel in demanding service conditions.

## 1. Introduction

Depending on the degree of alloying, heat (or creep)-resistant steels can be classified as: (i) unalloyed steels, (ii) alloyed (Mo, CrMo, and CrMoV) steels, and (iii) alloyed austenitic (Cr-Ni and Cr-Ni-Mo) steels. The service temperature of these steels increases with the complexity of the chemical composition, so high-alloyed steels can be used at operating temperatures exceeding 600 °C with significantly higher creep strength compared to low-alloyed steels, which enables reduced wall thicknesses for components under the same operating conditions they are exposed to. Still, despite their resistance to high temperatures, high-alloyed heat-resistant steels can also be exposed to operating conditions (predominantly in pressure vessel equipment) that can lead to microstructural changes and material degradation (caused additionally by excessive preheating during post-weld heat treatment (PWHT)). This was investigated by Jovanović et al. [1], who studied changes in martensitic lath width that affect the reduction of mechanical properties [2] as well as creep cracking [3,4].

The initial task of welding and repair welding is to meet strict requirements regarding mechanical properties, which furthermore provide safe and reliable service not only for the structure itself but for the whole facility as well. It sounds like an easy task, but experience and in-field conditions impose a different reality, in addition to the complexity of metallurgical properties that come with high-alloyed heat-resistant steels and their welding properties. This mainly reflects one of the most-investigated topics regarding this group of steel—their creep resistance after welding [5,6]. Zeman et al. [7] pointed out the importance of adequate heat treatment of martensitic steel welded tubes by emphasizing how dangerous cracks in heat-affected zones (HAZs) can be. Meanwhile, some authors use the structural integrity approach to verify the quality of welded joints made of different types of materials, such as [8,9].

The heat-resistant steel P92 (also known in the literature as 1.4901/X10CrWMoVNb9-2/ASTM A335 P92) has been developed as a suitable material for power plant applications that operate at elevated temperatures, precisely in the temperature range of 620–650 °C. This steel represents a high-temperature martensitic steel from the group of 9–12% CrMoV content group of creep-resistant steels. Numerous studies have dealt with some basic frameworks regarding the mechanical properties and application limit [10,11] specifics of steel P92, particularly concerning its weldability [12,13] and weldability to other materials, especially to newly developed austenitic steels [14], as well as the problem of residual stress in heterogeneous welded joints [15]. Special attention was put on the creep rupture problem of heterogeneous welds made of steel P92 and other materials [6,16,17,18,19], emphasizing the fact that they represent the weakest spot in construction. Most research has relied on microstructure analysis to determine the degradation level of this steel after long service periods—such as the work by Yangyang et al. [20], Duan et al. [21], and Zielinski et al. [22] focusing on mechanical properties—or has been oriented towards the oxidation resistance of their coatings, such as Zhou et al. [23]. Creep resistance also represents one of the favorite topics of investigation for P92 steel, such as the paper by Kral et al. [24], whose authors investigate the influence of high-pressure sliding and rotary swaging on this problem, while a few interesting summaries regarding creep properties can be found in [25,26].

Welded joints made of P92 steel pose a considerable challenge to structural integrity assessment due to their propensity for type IV cracking, i.e., cracks occurring in the HAZ [27,28,29]. Furthermore, the literature discusses residual stress in welded joints of this steel [30], though this is not within the scope of this paper. Consequently, these described factors can lead to a decrease in the operational life of components, which can be attributed to:

(a) Operational conditions exceeding design limits;
(b) Inadequate structural design and poor material selection; and
(c) Extensive repair welding procedures that introduce excessive heat and cause subsequent material degradation.

Material properties and their microstructure play vital roles in each of these contributing factors. Given the inherent complexity of high-alloyed heat-resistant steels, their welding represents a critical aspect affecting structural integrity. Consequently, the development of a non-uniform microstructure across P92 weldments renders their weldability a complex undertaking that needs careful consideration.

The mechanical properties and microstructure of welded P92 steel are largely determined by the preheating, heat input during welding, interpass temperature, and PWHT. However, the consequences of subsequent repair welding, involving additional preheating, heat input due to repair welding, and PWHT, raise significant questions. Specifically, what microstructure would be expected in the parent material, what microstructure would be expected in HAZ, and how would mechanical characteristics change across each welding joint zone after repair welding?

This paper presents an ongoing investigation into the mechanical characteristics and microstructure of both initially welded and repair-welded heat-resistant steel P92. Welding and repair welding were performed on a pipe with an outer diameter of Ø280 mm and a butt-welded U-groove joint configuration (hereafter referred to as BW-U). The investigation encompassed three welded pipe cases: two initially welded pipes (one in the PF–vertical upwards position, and the other in the PC–horizontal–vertical position) and one PF-welded pipe subjected to a simulated repair welding procedure, also in the PF position. The welded joint in the PC position is considered critical and particularly worthy of analysis due to the higher heat input compared to the PF welded case. Furthermore, it is important to note that repair welding often occurs under field conditions—i.e., in non-ideal environments for welding activities—where complete removal of the original weld metal (WM) is often impractical or infeasible. This aspect is given special consideration throughout the manuscript. Detailed welding and repair welding technologies are also provided, although they are not the primary focus of this paper. A dedicated subchapter outlines the fundamental characteristics of P92 steel. A comparative analysis of each welding case (PF welded, PC welded, and PF repair welded) and each welding joint zone (PM/HAZ/WM) was conducted by evaluating their mechanical characteristics (uniaxial tensile properties, impact toughness, and micro-hardness) and through microstructure analysis. All tests were performed at room temperature, with sampling and testing procedures adhering to relevant standards (described in the manuscript). The presented analysis provides insight into the mechanical and microstructural properties of the welded joint zones and the general behavior of creep-resistant P92 at room temperature. The influence of heat input from the thermal cycles during the initial and repair welding procedures was analyzed to further elucidate the application limits of high-alloyed heat-resistant steel P92.

## 2. Heat-Resistant Steel P92

Heat-resistant steel P92 is an improved P91/T91/F91 steel grade, characterized by an approximate 2% addition of W, with a slightly increased Cr content, and a reduced Mo content. While P92 steel exhibits a lower yield strength compared to P91 steel at room temperature, its tensile strength surpasses that of P91 under the same conditions. Notably, its primary advantage lies in its superior creep resistance, which is approximately 30% higher at temperatures of up to 600 °C compared to P91. This improvement is attributed to the increased W content, which enhances the strengthening mechanisms within the martensitic microstructure. Furthermore, this steel grade demonstrates improved corrosion resistance and weldability compared to P91, making it widely adopted in supercritical power generation units. Additionally, it offers significantly high-temperature strength and oxidation resistance. These properties render it highly suitable for applications in high-temperature and high-pressure environments where other materials may experience premature failure or degradation. It can withstand service temperatures up to 650 °C and possesses a minimum yield strength of 440 MPa. This high-alloy martensitic steel finds primary application in power plant facilities, petrochemical facilities, and oil refineries—predominantly within the energy sector due to its advantageous characteristics.

The chemical composition and tensile properties of two batches of hot-rolled creep-resistant steel P92 (according to the inspection documents defined by standard EN 10204 [31] and technical delivery conditions by EN 10216-2 [32]) used for welding are presented in Table 1 and Table 2, respectively. Each batch is used for one pipe side.

Due to the chemical similarities between P92 and P91 steels, their metallurgical properties are also comparable. The same parameters and elements that contribute to the strength of steel P91 are responsible for the strength of steel P92 grade, which is deeply connected to the microstructure. Similarly, the microstructure exhibits sensitivity to the material’s fabrication environment, chemical composition, heat treatment/heat input, and cold working. It is important to note that if P92 steel undergoes PWHT at a sufficiently low temperature, it can become susceptible to stress corrosion cracking. Subsequently, the obtained martensitic structure requires further tempering and residual stress reduction via heat treatment (i.e., annealing) at approximately 720–780 °C. Cracking due to stress corrosion can occur in tempered martensite; therefore, it is recommended that PWHT be performed immediately as a consequence of rapid cooling to 300 °C, followed by slow cooling in the air. Furthermore, heat-resistant steel P92 can also be susceptible to stress corrosion cracking in its as-welded condition; therefore, the duration between the welding passes and its subsequent heat treatment (particularly in humid conditions) must be optimized. Otherwise, its susceptibility to cracking increases rapidly. Consequently, all these factors determine the thermal cycle during welding, ensuring a desirable microstructure in each welding zone, which leads to satisfactory mechanical properties and, ultimately, the reliable and safe operation of the equipment in general.

This steel necessitates strict technical specifications regarding preheating, welding current, interpass temperature, weld bead width, PWHT timeline, etc. The high content of alloying elements such as Cr, Mo, and W in P92 steel diminishes its weldability. Another challenge encountered during the welding of P92 steel pertains to the potential formation of δ-ferrite, which, in higher content, may lead to hot cracks [33]. This is attributed to the presence of a higher weight percentage of ferrite-stabilizing elements, including V, Nb, W, and Mo [9].

## 3. Welding and Repair Welding

As previously noted, welding of heat-resistant steel P92 presents a significant challenge due to strict technical requirements encompassing preheating temperature, welding current, interpass temperature, bead width, and PWHT. These specific requirements are due to the high content of alloying elements, specifically Cr, Mo, and W, which significantly affects its weldability. Considering these factors, the Gas Tungsten Arc Welding (GTAW) and Shielded Metal Arc Welding (SMAW) processes were selected for the root pass and subsequent fill passes, respectively. The modular welding machine EWM Tetrix XQ 300 puls DC Comfort 3.0 8P (Mündersbach, Germany) is used for both welding processes, since it supports both welding regimes. It also refers to repair welding. Due to specific requirements concerning heat input as well as welding position, welding is carried out manually by certified staff.

**Figure 1:**
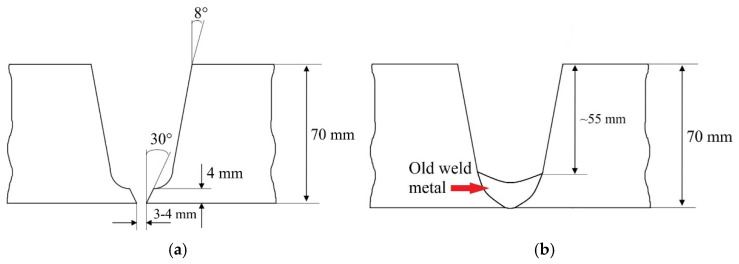
BW-U grooves geometry of (**a**) pipe No. 1 and No. 2; (**b**) pipe No. 3 meant for repairing.

In all three cases, the pipes were 70 mm thick, with an outer diameter of Ø280 mm and a butt-welded U-groove joint configuration (BW-U) as depicted in Figure 1a. The preparation of the groove on the (repaired) pipe involved machining, specifically grinding, to remove the majority, but not all, of the existing WM zone, as illustrated in Figure 1b. This particular groove preparation simulated a repair welding under conditions of limited resources, mobility, and accessibility to damaged areas.

**Figure 2:**
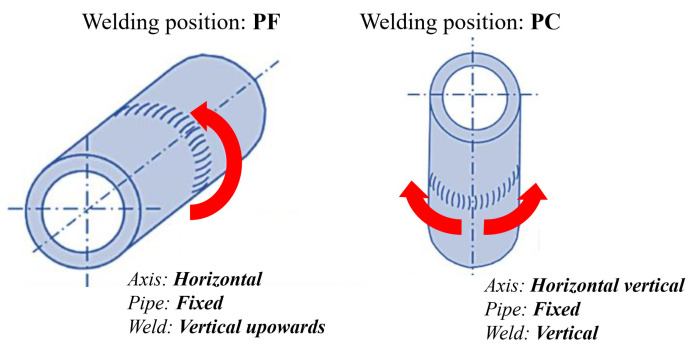
PF and PC welding positions.

The original two welding joints on pipes were made using the PF (vertical upwards) and PC (horizontal–vertical) welding positions (Figure 2). A repair welding simulation was then conducted on the PF-welded pipe, also in the PF position, requiring different welding techniques and operator skill. Welding position dictates heat input, with the PC position representing a critical case due to the higher heat input needed for adequate fill. Table 3 provides a summary of the welding samples, processes used, and other relevant data.

In all three welding probes (original welding and repair welding of the pipes), the selection of filler materials was based on the parent material’s metallurgical properties and the recommendations provided by the filler material manufacturer.

The specific characteristics of the heat-resistant P92 steel necessitate the utilization of filler materials/electrodes with an identical or similar chemical composition. The filler material employed for the GTAW welding process was a rod WZ CrMoWVNb 9 0.5 1.5 grade (commercially designated as Thermanit MTS 616 by Böhler Welding, Düsseldorf, Germany). This is a high-alloyed, creep-resistant GTAW rod specifically designed for high-temperature service and root passes. The resulting microstructure is martensitic, making it suitable for subsequent quenching and tempering heat treatments. The shielding gas used was Argon 99.996% with a flow rate of 8–10 L/min, while the backing gas needed to be used is also Argon 99.996% with a flow rate of 10–14 L/min. The electrode used for GTAW was a WT20 type with a diameter of Ø2.4 mm.

The SMAW welding process utilized the E ZCrMoWVNb 9 0.5 2 B 4 2 H5 covered electrode (commercially designated as Böhler FOX P 92 by Böhler Welding). This high-alloy, creep-resistant basic electrode is specifically designed for welding P92 creep-resistant steel, offering a stable arc, low spatter, and easy slag removal. It was suitable for continuous use up to +650 °C.

The chemical compositions and tensile properties of both filler materials are provided in Table 4 and Table 5, respectively.

During welding, steel P92 is exposed to the welding in the martensitic range, which is a direct consequence of its microstructure.

**Figure 3:**
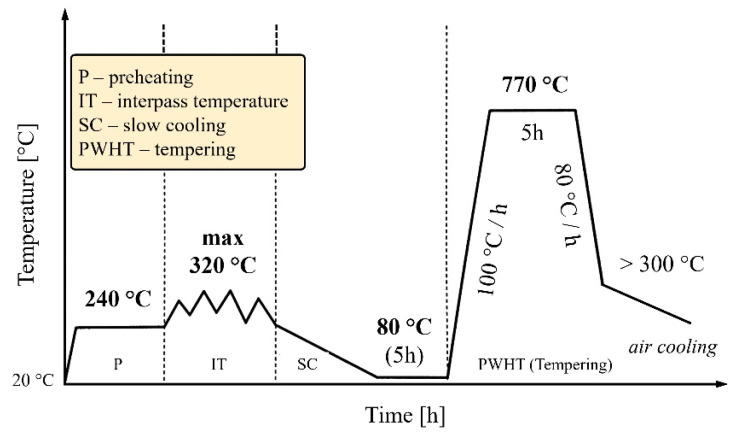
Thermal cycle welding and repair welding of P92.

The thermal cycle for both welding and repair welding of pipes made of creep-resistant steel P92 is given in Figure 3, adopted based on all features and pipe geometries, as well as the material itself. Preheating of all pipes was performed using resistance heaters mounted on both sides of the pipes to be welded, according to the recommended thermal cycle for steel P92 (Figure 3). This involved maintaining a preheating temperature up to 240 °C. Upon cooling, the weld metal immediately transforms into martensite, which is tempered by the heat input during each subsequent welding pass. These same resistance heaters were used to control the interpass temperature between welding passes at ~320 °C. Immediately following welding, and without removing the resistance heaters from the pipes, a PWHT was conducted by reaching a temperature of ~770 °C and holding it for a defined duration of 5 h. Subsequently, the heaters were turned off and removed for the purpose of rapid cooling down to ~300 °C, followed by slow (air) cooling.

The welding parameters for all welding procedures (both PF1 and PC2 and the repaired PF3 pipe) are detailed in Table 6, Table 7 and Table 8, with clear identification of each pass designation and the corresponding welding parameters, including values for current, voltage, polarity, and heat input. Travel speed (of welding) has varied in the range 60–90 mm/min, which dictates heat input values not to exceed 1.8 kJ/mm. It is important to emphasize that the initial welding parameters applied to pipe PF3 were identical to those used for pipe PF1.

All welded joints were subjected to non-destructive testing, adhering to the EN ISO 15614-1 [34] standard. In this particular case, visual testing, penetrant testing, and ultrasonic testing were performed in order to detect potential welding defects that may affect the testing results. No defects were observed.

## 4. Experimental Testing

**Figure 4:**
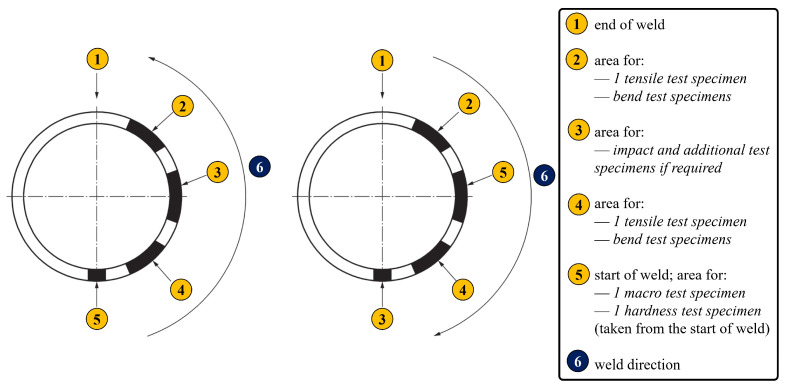
Sampling scheme for testing specimens for butt welded joint in pipe.

The results of the experimental testing performed on both the (i) PF- and PC- welded and (ii) PF repair-welded pipes are presented in this section. This testing included (1) tensile testing, (2) impact testing, and (3) hardness measurements. The results of the microstructural analysis are given in a specific section, with an overview of the obtained experimental results related to the mechanical properties. Test specimens for destructive evaluation (tensile and impact testing), as well as for hardness assessment and metallographic analysis, were sampled from both types of pipe following the EN ISO 15614-1 standard according to the sampling scheme given in Figure 4. These evaluations provided a solid base for understanding the properties of both the original and repaired welded joints in the welded pipes, with particular attention paid to each welding zone, indicating their attributes and potential service restrictions.

### 4.1. Tensile Testing

**Figure 5:**
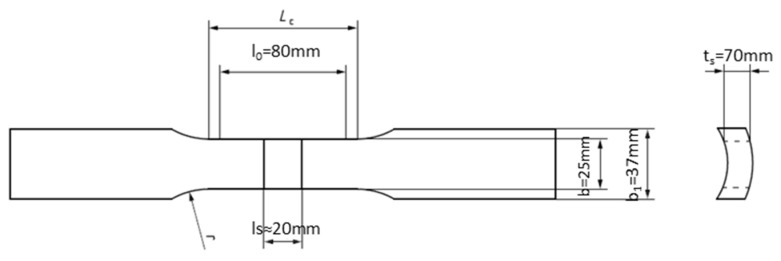
Tensile test specimen geometry.

Following the recommendations outlined in EN ISO 15614-1 standard, two tensile test specimens were taken in a direction perpendicular to the welded joint from each pipe. Specimens were prepared from both the new welded (PF and PC) joints and the repair-welded (PF) joint. Gauge length includes WM (central part of the specimen) and HAZ and PM on both sides of it. The technical drawing of the tensile specimen, defined in standard EN ISO 4136 [35], is given in Figure 5. The tensile testing procedure was conducted at room temperature with a testing rate of 5mm/min, adhering to the criteria of the EN ISO 4136 and EN ISO 6892-1 [36] standards. Testing was performed on ZWICK ROELL Z1200E, a tensile testing machine (ZwickRoell GmbH & Co. KG, Ulm, Germany) with a capacity of 1200 kN.

Two specimens were tested from each pipe, and the results, which include yield stress (R_p0.2_), tensile strength (R_m_), elongation, fracture location, and weld position, are given in Table 9.

### 4.2. Charpy Impact Testing

**Figure 6:**
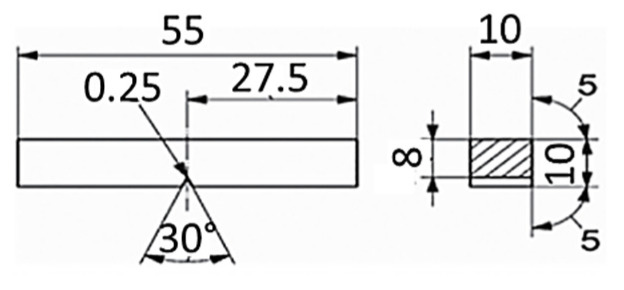
Charpy V-notch specimen geometry.

**Figure 7:**
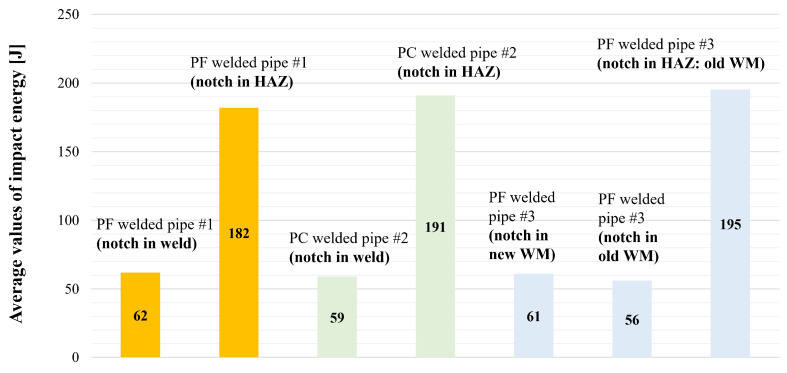
Average values of impact energy [J] by critical welded region in all three cases.

Impact toughness testing was conducted following the EN ISO 9016 [37] guidelines and EN 10216-2 acceptance criteria at room temperature, using an A. J. Amsler Schaffhausen Charpy Impact testing machine (Zürich, Switzerland) with maximum energy of 300 J. The Charpy impact test specimens (10 × 10 × 55 mm) had neck cross-sectional geometry of 10 × 8 mm, with the V-notch machined at critical locations within the welded joints (technical drawing shown in Figure 6). These critical locations encompassed the WM and HAZ for all three welded joints cases, along with the old WM (or its leftover) and HAZ regions for the repaired PF3. From each of the tested welded joints and their 2 two critical zones (WM and HAZ in PF1 and PC2, new and old WM and HAZ in PF3), 3 specimens were made—in total, 21 specimens. Results are shown in Figure 7. It should be stated that even though the average impact toughness value of the PF3 sample is higher than the minimum defined by the parent material standard, one of the samples exhibited a value of 41 J, which is at the bottom limit.

### 4.3. Hardness Measuring

**Figure 8:**
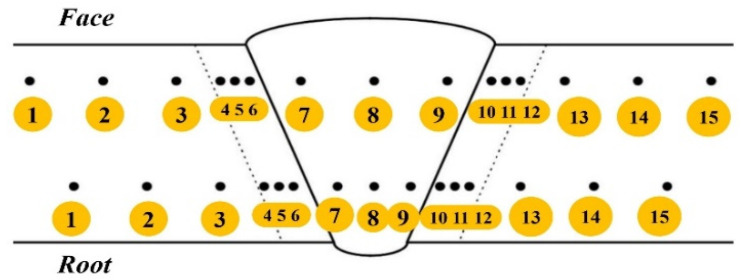
Locations of hardness measuring testing: proximal to the weld face and root.

Hardness measurements were conducted along upper and lower rows (proximal to the weld face and root, respectively), with 15 measurement points per row on both the originally welded and the repair-welded joints. These measurements covered both parent material, both HAZs, and the weld metal itself, as illustrated in Figure 8. Additionally, on the repair-welded joint (PF3), hardness measurements extended to the regions of the old (original) weld joint on the root side. Each testing zone of the welded joint comprised 3 measurement points. The measurement procedure adhered to ISO 6507-1 [38] standards, employing Vickers hardness testing with a test load of 10 kgf (HV10). The surface intended for measurement was etched using a 3% solution of nitric acid in alcohol (i.e., Nital solution). Hardness testing was performed on a Reicherter Stifelmayer 250H hardness testing device (Reicherter Prüftechnik GmbH, Esslingen am Neckar, Germany).

**Figure 9:**
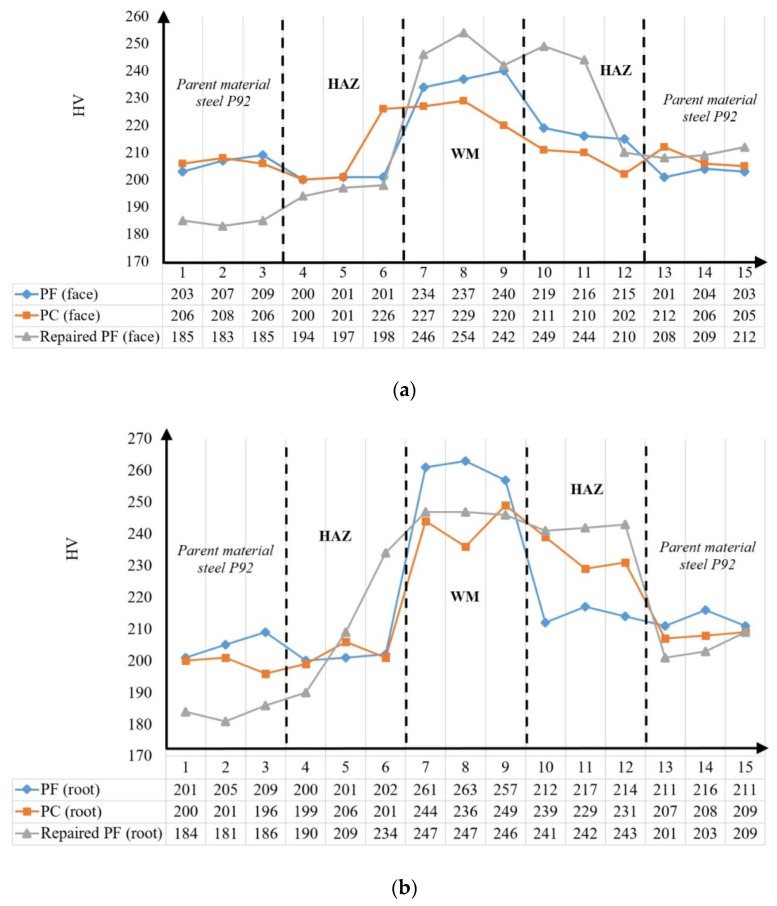
Hardness values and their comparison between zones/welding cases: (**a**) measuring line closer to face side; (**b**) measuring line closer to root side.

Figure 9a compares the hardness values obtained along the measurement traverse closer to the weld face, while Figure 9b shows the comparison for the traverse closer to the weld root.

## 5. Microstructural Analysis

Specimens for microstructural analysis were sampled according to the EN ISO 15614 standard recommendation. Samples were prepared by grinding on wet papers of different granulations (P240–P1200). Grinding was performed in such a way that each subsequent sandpaper removed traces of the previous one. Samples were polished afterward, with diamond pastes of 3 μm and 1 μm granulations. Metallographic samples were chemically etched by a 3% solution of nitric acid in alcohol (i.e., Nital solution). The metallographic analysis was performed on a Leica DM4 light microscope, which is equipped with a digital camera and appropriate Leica Las v 4.9 software. Microstructural analysis was made on each welded joint, i.e., the new (PF1 and PC2) welds and the repaired one (PF3), at the representative positions.

During microstructural analysis of the PF1 and PC2 newly welded joints, as well as the repaired PF3, each zone of the welded joint was examined in detail, including both parent materials (HAZ and WM), over the entire cross section. From a microstructural point of view, the welded joint in the PC position is considered to be critical due to the higher heat input compared to the PF welded case (i.e., caused by welding position). In that manner, microstructural analyses were performed on samples welded in the PC position for the new joint and in the PF position for the repair joint.

**Figure 10:**
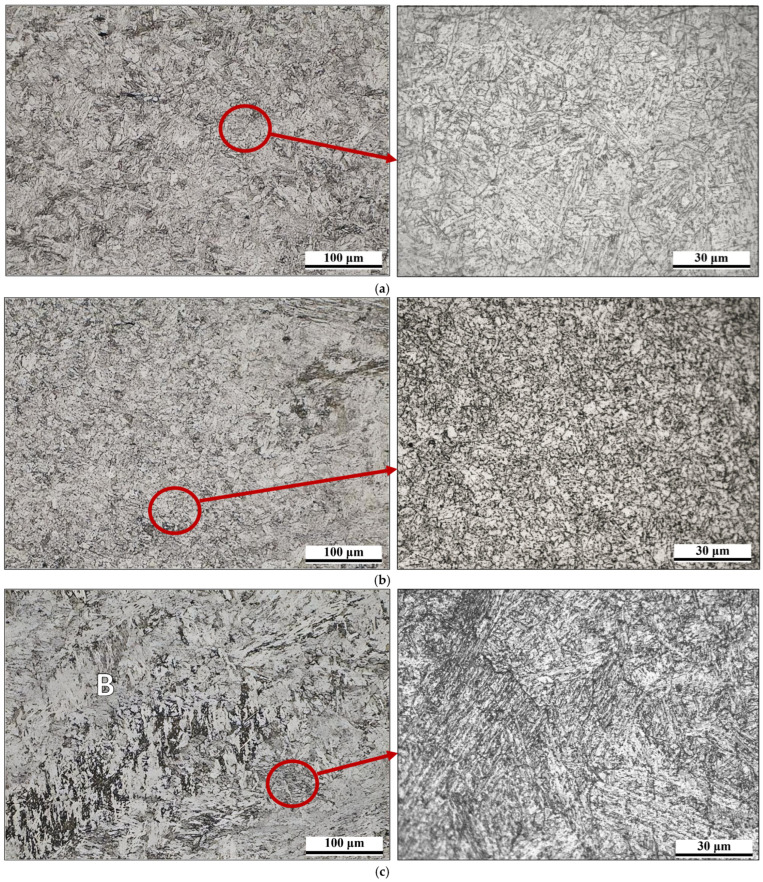
Microstructure of PC2 welded sample (**a**) PM, (**b**) HAZ, (**c**) WM.

Representative microstructures of parent material (steel P92) are given in Figure 10a with two magnifications. The microstructure of the parent metal is tempered martensite, which consists of martensite laths with precipitated carbides, with areas characterized by sets of parallel martensitic laths (Figure 10a). HAZ consists of a similar microstructure, but is characterized by finer martensitic laths, which were oriented in a more chaotic manner compared to those in the parent material, which is more obvious in the figure with higher magnification (Figure 10b). However, the microstructure of HAZ in the area close to the fusion line is characterized by some amount of coarse laths, along with coarse carbide particles. On the other hand, the microstructure of WM consists of tempered martensite as well as small amounts of bainite (designated as B), as shown in Figure 10c. It should be emphasized that the WM microstructure has a coarser microstructure compared to the microstructure of the parent material and HAZ.

**Figure 11:**
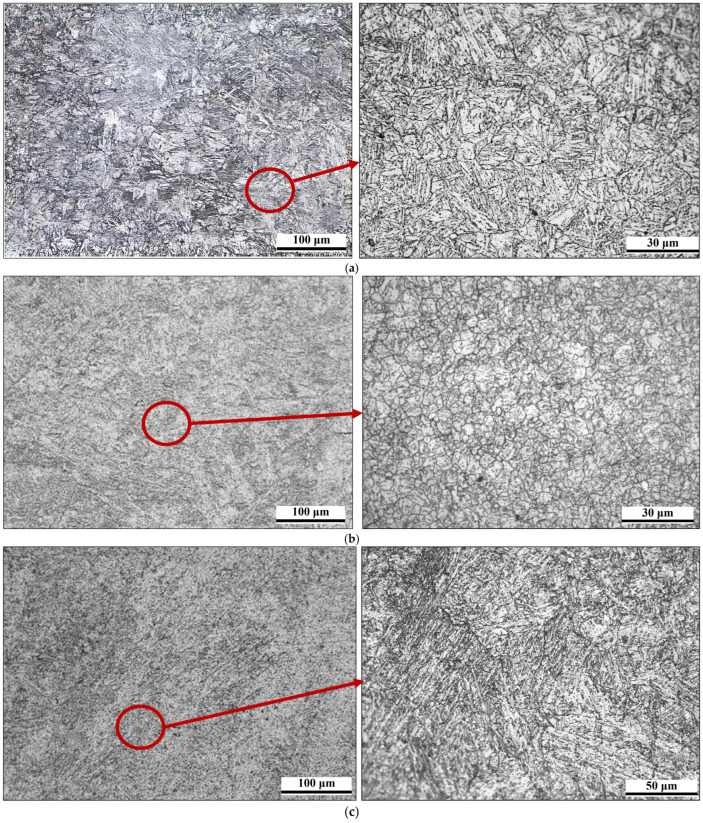
Microstructure of PF3 welded sample of (**a**) PM, (**b**) HAZ, (**c**) WM.

Regarding the PF3 repair welding pipe, the microstructure of the parent material dominantly consists of tempered lath martensite (Figure 11a). Compared to the PF1 welded joint, after the repair welding, the tempered martensite in the PM is characterized by larger second-phase particles (Figure 10a and Figure 11a). The HAZ areas in the welded joint are approximately 15–20% wider compared to the HAZ areas of the new joint, due to (additional) heat input. It should be emphasized that the microstructure of HAZ is also characterized by tempered martensite with coarser carbide particles compared to the HAZ in the new PF welded joint, as shown in Figure 10b and Figure 11a. Regarding the WM, its microstructure consists of coarse tempered martensite (Figure 11c).

## 6. Discussion

In both initially welded (PF1 and PC2) and repair-welded (PF3) joints, the WM and HAZ consistently exhibited higher hardness values compared to the PM, which is primarily attributed to the formation of martensite microstructures in these regions caused by the welding process.

The fracture in all tensile tested specimens occurred in the ‘weaker’ PM, which highlights the overmatching effect [39,40,41,42]. However, lower values of ultimate tensile strength (R_m_) were noted in the repaired PF3 pipe compared to the initially welded ones. This difference can likely be attributed to the repair process itself, as a direct consequence of the additional heat input due to repair welding and the PWHT. Note that in the case of the repair-welded pipe (PF3), the PM, HAZ, and the small leftover portion of WM were exposed to double heat input (due to welding/repair welding) and double PWHTs (due to the initial and repair welding process).

The results of the Charpy impact testing reveal a trend of superior impact toughness in the HAZ compared to the WM for the initially welded (both PF1 and PC2) pipes. This suggests that the thermal cycle experienced by the HAZ during the original welding process likely resulted in appropriate microstructural evolution. These changes in microstructure enhance its resistance to brittle fracture initiation and propagation under impact loading relative to the microstructure of the WM. Higher impact toughness values in HAZ could be associated with the notch root position in the fine-grain microstructure area. Therefore, the slight increase in impact toughness in the HAZ observed in the PC welded pipe compared to the PF welded one could be attributed to subtle differences in the welding thermal cycle associated with the welding position, potentially leading to a more favorable microstructural arrangement within its HAZ. Concerning the repaired PF3 pipe, the impact toughness of the newly formed WM mirrored that of the original WM. Even though the microstructures of each WM look alike, the low impact energy of one PF3 sample might be the consequence of welding defect(s) or a coarser microstructure caused by higher heat input due to additional passes made with the TIG process. However, the unexpectedly high impact toughness exhibited by the HAZ of the old WM in the repaired pipe demands further microstructural investigation. One hypothesis regarding its microstructure is that the subsequent thermal cycle from the repair welding and the PWHT may have produced a more homogeneous microstructure, potentially optimizing the size and distribution of carbides that might improve its impact energy absorption capacity.

The measured hardness values are intrinsically linked to the microstructures observed on the analyzed surfaces. Notably, higher hardness values were consistently observed in the WM region, followed by the hardness measured in the right-side HAZ across all three cases. This trend is attributed to the presence of tempered martensite microstructure [39]. No significant difference in hardness values was detected in the PM and HAZ for the PF1-welded pipe. However, in the case of the repaired pipe (PF3), slightly higher hardness values in the WM and the right-side HAZ were observed. This increase is a consequence of the heat input due to the repair welding and the subsequent PWHT, evident in both the face-side and root-side measurement traverses. This observation also applies to the PC2 pipe. Furthermore, the repair welding and associated additional thermal cycle have been shown to influence the hardness values of the PM when comparing the PC/PF-welded cases with the repaired ones.

An extensive investigation of the mechanical properties at room temperature offers preliminary input for a better understanding and characterization of the welding and repair welding features of P92 steel. This work poses a critical question: How will elevated temperatures influence the microstructure evolution of each welded joint zone, and thus their mechanical properties?

The results of this study offer/suggest:

- Appropriate welding and repair techniques with recommended welding parameters, leading to defining an appropriate welding procedure specification (which has to be carried out at room temperature).
- The aforementioned led to satisfactory mechanical properties of the welds at room temperature, supported by favorable microstructures within each welded joint zone for every welded pipe case. Nevertheless, the effect of heat input (due to welding or post-weld heat treatment) on these properties, particularly concerning the WM and HAZ, warrants a comprehensive investigation at higher temperatures.
- Identification of potentially critical spots during the welding of P92 steel and subsequent repair welding, based on an analysis of mechanical properties and metallographic analysis.
- The necessity of utilizing more advanced techniques for full characterization of each welded joint zone, which is crucial for elevated temperature applications. This predominantly refers to metallographic analysis.

## 7. Conclusions

This study emphasizes the necessity of thorough microstructural characterization to understand and predict the behavior of WM and HAZ during the welding of heat-resistant steel P92, particularly in complex scenarios involving repair welding. This characterization is crucial for ensuring the reliability and safety of welded structures made of heat-resistant steel P92.

The experimental findings underscore the potential for repair welding to cause significant changes in the existing WM and HAZ microstructure, which can affect the integrity of the repaired joint. One key recommendation regarding the repair welding technique for steel P92 drawn from this research is to strictly define the heat input—for instance, by making more welding passes with the GTAW process. This is a precaution against brittle fracture and δ-ferrite formation.

Overall, the research contributes valuable insights into optimizing welding and repair welding procedures for P92 steel by providing a deeper understanding of the interplay between welding parameters, microstructure, and mechanical properties.

Therefore, based on the presented results, the following conclusions can be drawn:

- Tensile testing revealed that fractures occurred consistently in the parent material, indicating an overmatching effect.
- The repaired (PF3) welded joints have lower ultimate tensile strength compared to the PF1 and PC2 welded ones, which can be attributed to microstructural changes induced by the additional heat input during the repair welding and PWHT, affecting the tempering of the martensite.
- Impact testing demonstrated that specimens with the notch in the HAZ exhibited higher impact energy values, suggesting that specific microstructural features within the HAZ (likely related to grain refinement or tempering effects) contribute to improved toughness compared to the WM. Another thermal cycle due to repair welding also contributes to this statement, since the average impact energy of repaired welded pipe is higher than both original welded ones.
- In the repair-welded case, a slight increase in hardness was noted in the WM and HAZ, likely due to the supplementary heat input and PWHT contributing to further microstructural changes.

Considering the limitations highlighted in the Section 6, the next phase of investigation will involve more detailed qualitative and quantitative metallographic analysis. The aim is to fully understand the welding/repair welding features of P92 steel and its weld zones, especially at elevated temperatures, utilizing advanced material characterization techniques. Additionally, a fracture mechanics approach will serve as an analytical method for a potential structural integrity assessment model for each welded joint region. This will link the obtained mechanical properties, providing reliable limitations in engineering practice.

## Figures and Tables

**Table 1:** Chemical composition of steel P92 [wt%].

	C	Mn	Si	S	P	Nb	Cr	Ni	Al	V	Mo	W	B	Cu	N	Fe
Batch 1	0.119	0.42	0.166	0.003	0.009	0.054	8.75	0.14	0.005	0.194	0.5	1.55	0.016	0.14	0.053	bal.
Batch 2	0.114	0.41	0.158	0.003	0.011	0.055	8.6	0.23	0.004	0.192	0.49	1.52	0.002	0.143	0.05	bal.

**Table 2:** Mechanical properties of steel P92 (at room temperature).

	R_e_ [MPa]	R_m_ [MPa]	A5 [%]
Batch 1	580	728	21
Batch 2	514	683	28

**Table 3:** Designation and geometry of pipes prepared for welding, with welding position and joint type.

Pipe Designation	Welding Position	Welding Process	Thickness [mm]	Outside Diameter [mm]	Joint Type	Remark
**PF1**	PF	GTAW/SMAW	70	280	BW-U	*Welding*
**PC2**	PC	GTAW/SMAW	70	280	BW-U	*Welding*
**PF3**	PF	GTAW/SMAW	70	280	BW-U	*Repair welding*

**Table 4:** Chemical composition of filler materials WZ CrMoWVNb 9 0.5 1.5 and E Z CrMoWVNb 9 0.5 2 B 4 2 H5 [wt%].

Filler Material/Element	C	Mn	Mo	Si	Cr	Ni	W	Nb	V	N	Fe
**W Z CrMoWVNb 9 0.5 1.5**	0.1	0.5	0.4	0.25	8.5	0.5	1.6	0.06	0.2	0.04	*bal.*
**E Z CrMoWVNb 9 0.5 2 B 4 2 H5**	0.1	0.7	0.55	0.3	8.6	0.7	0.06	0.04	0.2	0.04	*bal.*

**Table 5:** Mechanical properties of filler material filler materials of WZ CrMoWVNb 9 0.5 1.5 and E ZCrMoWVNb 9 0.5 2 B 42 H5 guaranteed by the manufacturer (at room temperature) after PWHT (annealed, 760 °C/2 h; cooling down to 300 °C/air).

Filler Material	Re [MPa]	Rm [MPa]	A (L_0_ = 5d_0_) [%]	Impact Fracture [J]
**W Z CrMoWVNb 9 0.5 1.5**	560	720	>15	>41
**E Z CrMoWVNb 9 0.5 2 B 4 2 H5**	600	740	20	>55

**Table 6:** Welding parameter (PF1).

Pass	Process	Filler Metal Diameter [mm]	Current [A]	Voltage [V]	Type of Current/ Polarity	Heat Input [kJ/mm]
Root 1	GTAW	2.4	95–105	13–15	DC-	~0.84
Fill 2–3	GTAW	2.4	125–135	13–15	DC-	~1.05
4–5	GTAW	2.4	135–145	13–15	DC-	~1.17
6–8	SMAW	2.5	70–80	21–23	DC+	~1.3
9–11	SMAW	2.5	80–95	21–23	DC+	~1.4
12–14	SMAW	2.5	80–95	21–23	DC+	~1.4
15–17	SMAW	3.2	100–105	21–23	DC+	~1.5
18–20	SMAW	3.2	100–110	21–23	DC+	~1.5
21–23	SMAW	3.2	110–120	21–23	DC+	~1.5
24–27	SMAW	3.2	105–125	21–23	DC+	~1.5
28–31	SMAW	3.2	110–125	21–23	DC+	~1.5
32–35	SMAW	3.2	115–125	21–23	DC+	~1.55
36–39	SMAW	3.2	115–125	21–23	DC+	~1.5
40–43	SMAW	4.0	120–135	21–23	DC+	~1.6
44–48	SMAW	4.0	120–135	21–23	DC+	~1.6
49–53	SMAW	4.0	120–135	21–23	DC+	~1.5
54–58	SMAW	4.0	115–135	21–23	DC+	~1.6
59–62	SMAW	3.2	115–135	21–23	DC+	~1.6
63–67	SMAW	4.0	130–135	21–23	DC+	~1.6
68–72	SMAW	3.2	110–115	21–23	DC+	~1.5
73–77	SMAW	3.2	110–115	21–23	DC+	~1.4
78–82	SMAW	3.2	110–115	21–23	DC+	~1.4
83–87	SMAW	3.2	110–115	21–23	DC+	~1.4
88–92	SMAW	2.5	80	21–23	DC+	~1.4
Cover 93–98	SMAW	2.5	80–85	21–23	DC+	~1.3

**Table 7:** Welding parameter (PC2).

Pass	Process	Filler Metal Diameter [mm]	Current [A]	Voltage [V]	Type of Current/Polarity	Heat Input [kJ/mm]
Root 1	GTAW	2.4	100–110	13–15	DC-	~0.9
Fill 2–4	GTAW	2.4	140–145	13–15	DC-	~1.1
5–7	GTAW	2.4	140–145	13–15	DC-	~1.1
8–11	SMAW	2.5	80–90	21–23	DC+	~1.3
12–15	SMAW	2.5	85–95	21–23	DC+	~1.3
16–19	SMAW	2.5	90–100	21–23	DC+	~1.35
20–23	SMAW	3.2	110–120	21–23	DC+	~1.5
24–27	SMAW	3.2	115–125	21–23	DC+	~1.5
28–31	SMAW	3.2	115–125	21–23	DC+	~1.5
32–35	SMAW	3.2	115–125	21–23	DC+	~1.5
36–39	SMAW	3.2	120–130	21–23	DC+	~1.6
40–43	SMAW	3.2	120–130	21–23	DC+	~1.6
44–47	SMAW	3.2	120–130	21–23	DC+	~1.6
48–51	SMAW	4.0	140–150	21–23	DC+	~1.7
52–56	SMAW	4.0	140–150	21–23	DC+	~1.6
57–61	SMAW	4.0	140–150	21–23	DC+	~1.7
62–66	SMAW	4.0	140–150	21–23	DC+	~1.6
67–71	SMAW	4.0	140–150	21–23	DC+	~1.7
72–76	SMAW	4.0	130–135	21–23	DC+	~1.6
77–82	SMAW	3.2	120–130	21–23	DC+	~1.4
83–88	SMAW	3.2	125	21–23	DC+	~1.4
89–94	SMAW	3.2	125	21–23	DC+	~1.5
95–100	SMAW	4.0	140–150	21–23	DC+	~1.6
Cover 101–109	SMAW	2.5	80–90	21–23	DC+	~1.3

**Table 8:** Repair welding parameter (PF3).

Pass	Process	Filler Metal Diameter [mm]	Current [A]	Voltage [V]	Type of Current/Polarity	Heat Input [kJ/mm]
Root 1	GTAW	2.4	85–95	13–15	DC-	~0.6
Fill 2–4	GTAW	2.4	100–110	13–15	DC-	~0.9
5–7	GTAW	2.4	115–125	13–15	DC-	~1
8–10	GTAW	2.4	125–135	13–15	DC-	~1.1
11–13	GTAW	2.4	140–150	13–15	DC-	~1.1
14–17	GTAW	2.4	145–155	13–15	DC-	~1.15
18–21	GTAW	2.4	145–155	13–15	DC-	~1.1
22–25	GTAW	2.4	145–155	13–15	DC-	~1.15
26–29	GTAW	2.4	190–200	13–15	DC-	~1.4
30–33	GTAW	2.4	190–200	13–15	DC-	~1.4
34–36	SMAW	3.2	120–130	21–23	DC+	~1.6
37–39	SMAW	3.2	120–130	21–23	DC+	~1.6
40–42	SMAW	3.2	120–130	21–23	DC+	~1.5
43–46	SMAW	3.2	120–130	21–23	DC+	~1.5
47–50	SMAW	3.2	120–130	21–23	DC+	~1.5
51–54	SMAW	3.2	120–130	21–23	DC+	~1.5
55–58	SMAW	3.2	120–130	21–23	DC+	~1.6
59–62	SMAW	3.2	120–130	21–23	DC+	~1.6
63–66	SMAW	3.2	120–130	21–23	DC+	~1.5
67–70	SMAW	3.2	120–130	21–23	DC+	~1.5
71–75	SMAW	3.2	120–130	21–23	DC+	~1.5
76–80	SMAW	3.2	120–130	21–23	DC+	~1.6
81–85	SMAW	3.2	120–130	21–23	DC+	~1.6
86–90	SMAW	3.2	120–130	21–23	DC+	~1.5
Cover 91–96	SMAW	2.5	80–90	21–23	DC+	~1.1

**Table 9:** Tensile testing results.

Specimen Label	R_p0.2_ [MPa]	R_m_ [MPa]	A [%]	Z [%]	Fracture Location
PF1-1	498	682	16	69	parent material
PF1-2	474	678	19	70	parent material
PC2-1	498	684	17	68	parent material
PC2-2	474	678	18	71	parent material
PF3-1	464	637	17	73	parent material
PF3-2	472	632	17	73	parent material

## Data Availability

The original contributions presented in this study are included in the article. Further inquiries can be directed to the corresponding author.
